# Clinical Effects of Zinc-containing Stents on Gingivitis: A Randomised Controlled Trial

**DOI:** 10.3290/j.ohpd.c_2275

**Published:** 2025-09-26

**Authors:** Bahar Alkaya, Hamza Gokhan Kayhan, Furkan Demirbilek, Mustafa Sahin, Nazli Totik, Mustafa Ozcan, Wim Teughels

**Affiliations:** a Bahar Alkaya Associate Professor, Department of Periodontology, Çukurova University, Adana, Turkey. Study conception and design, performed all clinical procedures, and revised the manuscript.; b Hamza Gokhan Kayhan Research Assistant, Department of Periodontology, Çukurova University, Adana, Turkey. Data collection, prepared and revised the manuscript.; c Furkan Demirbilek Research Assistant, Department of Periodontology, Çukurova University, Adana, Turkey. Data collection, prepared and revised the manuscript.; d Mustafa Sahin Research Assistant, Department of Orthodontics, Çukurova University, Adana, Turkey. Prepared the stents, revised the manuscript.; e Nazli Totik Research Assistant, Department of Biostatistics, Faculty of Medicine, Çukurova University, Adana, Turkey. Data analysis, prepared and revised the manuscript.; f Mustafa Ozcan Associate Professor, Department of Periodontology, Çukurova University, Adana, Turkey. Study conception and design, revised the manuscript.; g Wim Teughels Professor, Department of Oral Health Sciences, KU Leuven & Dentistry (Periodontology), University Hospitals Leuven, Leuven, Belgium. Study conception and design, revised the manuscript.

**Keywords:** dental plaque, gingivitis, stent, Zn, oral health

## Abstract

**Purpose:**

This study investigates the effects of zinc-containing stents on gingival inflammation, bleeding, and plaque regrowth in patients with gingivitis.

**Methods:**

A randomised, double-blind, placebo-controlled study was conducted at Çukurova University, enrolling 42 systemically healthy gingivitis patients aged 18–30. Participants were assigned to either a test group (Zn group) or a control group (placebo stents) and instructed to wear their stents for at least 12 h daily for 4 weeks after scaling. Clinical measurements, including gingival index (GI), plaque index (PI), and bleeding on probing (BOP), were assessed at baseline and at the 2nd, 4th, and 8th weeks. Statistical analysis was performed using IBM SPSS and RStudio.

**Results:**

Both groups showed statistically significant improvements in gingival health over time, but the zinc-stent group exhibited statistically significantly lower GI scores at all time points, suggesting a greater reduction in gingival inflammation. PI and BOP scores also improved in both groups, though no statistically significant difference was observed between them.

**Conclusion:**

The results indicate that zinc-containing stents may serve as a beneficial adjunct to mechanical plaque control in gingivitis management. Zinc’s antimicrobial and anti-inflammatory properties likely contribute to improved gingival health. The findings suggest that zinc-containing stents provide additional benefits in reducing gingival inflammation beyond mechanical plaque removal alone. These stents may be a promising adjunctive approach in periodontal therapy, but further long-term studies are needed to confirm their broader clinical applications.

**Trial registration:**

The study was conducted in accordance with the CONSORT guidelines and registered at clinicaltrials.gov (NCT06888440), 20 March 2025.

Gingivitis is a critical pre-condition for periodontal disease and one of the most common inflammatory diseases, affecting millions of individuals worldwide.^14^ It is widely regarded as a site-specific inflammatory disorder marked by gingival redness and swelling with no loss of periodontal attachment, mainly caused by the accumulation of dental biofilm.^3,22,31^ Host response is critical in periodontal diseases; untreated gingivitis can progress to periodontitis.^4,6^ Diagnosis is primarily based on clinical findings and periodontal parameters.^17^ Recent studies have highlighted the prominence of salivary biomarkers and genomics-based approaches for improving the diagnosis, risk assessment, and personalised management of periodontal diseases.^9,23^ In 2017, the American Academy of Periodontology (AAP) and the European Federation of Periodontology (EFP) concluded that the primary criterion for diagnosing gingivitis is ≥10%bleeding areas on probing, being considered localised when bleeding on probing (BOP) is between 10 and 30% and generalised when >30%.^25^ Predictions indicate an increasing prevalence of gingivitis, which is the primary risk factor for long-term tooth loss.^18^

Researchers have established mechanical plaque removal using a toothbrush and dental floss as an effective strategy for preventing gingivitis.^33^ The most common treatment method for gingivitis is mechanical dental plaque debridement and good oral care.^12^ Individuals, however, fail to maintain normal daily oral hygiene for various reasons, including brushing skills, oral construction, and others.^12^ In recent years, there has been a search for adjunctive therapy to help in treatment, in addition to the mechanical removal of dental plaque.^11^ Numerous studies show that antiplaque or anti-inflammatory agents have an additional effect on mechanical plaque removal.^16,21,26^ Additionally, recent studies have shown that vitamins can also be used as adjunctive agents in the prevention and treatment of gingivitis.^10^ Serrano et al showed that the use of antimicrobial agents resulted in better improvement in gingival, bleeding and plaque indices in their systematic review.^30^ However, the XIth European Workshop on Periodontology concluded that, while the use of chemical anti-plaque agents provides clear benefits in decreasing gingival indices in people with gingivitis, there is insufficient evidence to make firm recommendations on the use of anti-inflammatory agents.^11^

Zinc is one of the most abundant trace elements in the human body and plays a crucial role in numerous biological processes.  It helps the body absorb vitamins A, E, and folate and transforms many enzymes and proteins.^32^ Additionally, zinc plays a role in both adaptive immunity and natural immunity. It achieves this by regulating T-cell activity and by stimulating neutrophils and macrophages.^13^ Zinc is naturally used in plaque, saliva, and dental enamel hydroxyapatite in the mouth.It prevents the development of calculus, halitosis, and oral plaque.^5^ Antibacterial, anti-inflammatory, antioxidant, and healing activities are available. It is a strong antioxidant that reduces the production of toxic agents, such as hydrogen peroxide (H_2_O_2_), which have harmful effects on host cells.^37^ Zinc attaches to the oral bacterial surface and alters its surface potential, reducing bacterial adherence to teeth.^37^ Zinc supplementation is effective against different oral diseases such as gingivitis, periodontitis, and halitosis; zinc deficiency has been associated with poor oral and periodontal health.^5,32,36^ The safety of zinc compounds is the main benefit of zinc as a medicinal agent. It is used as toothpaste and mouthwash in oral health products to control and prevent the formation of dental plaque and dental calculus.^28^ Compared to conventional application methods such as mouth rinses and gels, intraoral stents may provide direct and controlled release of zinc to the target area, thereby prolonging the contact time and enhancing the local effect. In addition, the use of stents reduces the risk of the active substance spreading to undesired areas and minimises systemic absorption. Alkaya et al found that preoperatively, chairside made, zinc-containing surgical stents significantly benefit wound healing parameters and patients’ postoperative morbidity after free gingival graft harvesting.^2^ Similarly, Leventis et al found that the chairside fabrication of zinc-containing palatal stents for postoperative wound protection seems to constitute a valid, simple, time-saving, cost-effective clinical solution and offers significant benefits in post-operative bacterial control and enhancement of the early-phase palatal soft-tissue healing.^19^

To our knowledge, no previous randomised controlled trial has investigated the clinical efficacy of intraoral stents containing zinc as a localised delivery system for the management of gingival inflammation. Given the known anti-inflammatory properties of zinc and the recognised need for effective localised delivery systems in periodontal therapy, this randomised, double-blind, placebo-controlled clinical trial was conducted to investigate whether the adjunctive use of zinc-containing stents provides additional benefits in reducing gingival inflammation, bleeding, and plaque regrowth in patients with plaque-induced gingivitis.

## METHODS

This prospective, randomised, double-blind, and placebo-controlled study was conducted at the Department of Periodontology at Çukurova University between October 2023 and April 2024. The study, which was conducted following the ethical rules of the Declaration of Helsinki, was approved by the Ethics Committee of Çukurova University Faculty of Medicine (5 May 2023/133/55). The 41 patients included in the study were selected from individuals who were referred to the Periodontology clinic of Çukurova University Faculty of Dentistry and satisfied the criteria. Patients were informed and signed written informed consent before the study. The study was conducted in accordance with the CONSORT guidelines and registered at clinicaltrials.gov (NCT06888440).

### Sample Size

Since no study with the same hypothesis had been noticed before, the effect size of the study was 0.25 for four repeated measurements in two groups, with a type I error of 5% and a power of 95%, and the sample size with in-interaction ANOVA was calculated using G*Power 3.1.9.2. The study required at least 36 participants; however, because dropouts are common in studies, the estimated sample size was increased by 15% and identified as 42 (21 in each group).

Inclusion criteria:

Presence of plaque-induced gingivitis (bleeding on gentle probing at >30% of sites examined and a gingival index (GI) of at least 1 at >60% of sites examined);
Plaque index (PI) of ≥2 according to the modified Quigley&Hein index;
18–30 years old;
At least 20 natural teeth;
Systemically healthy with no physical or cognitive impairments.

Exclusion criteria:

Pocket probing depth (PPD) of ≥4 mm;
Interdental clinical attachment loss (CAL) detectable at ≥2 nonadjacent teeth or displaying buccal/oral CAL ≥3 mm coupled with PD ≥3 mm;
Subjects with a history of allergies to Zn;
The presence of haematologic disorders or any other systemic illness;
Pregnancy and breastfeeding;
Current orthodontic treatment;
History of periodontal therapy;
Use of antibiotics or anti-inflammatory medication within the preceding 6 months;
Smoking.

### Interventions

**Fig 1a to d:**
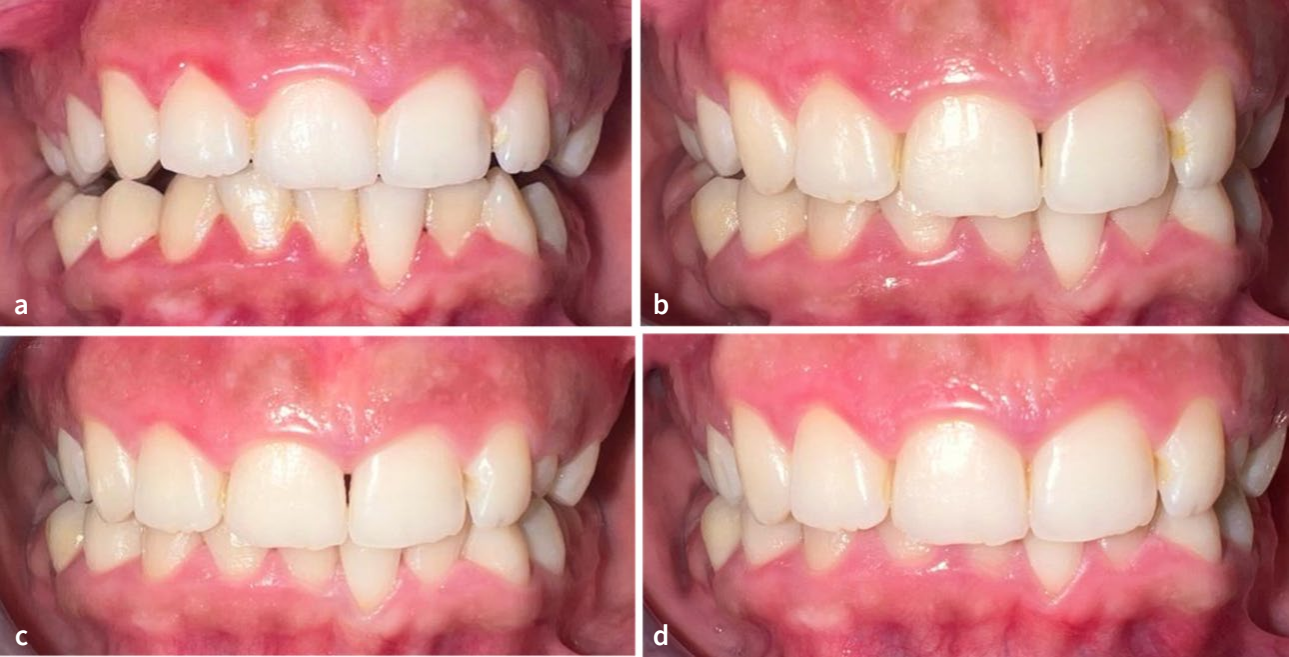
Clinical view of a patient who used Zn group: (a) baseline; (b) 2nd week; (c) 4th week; (d) 8th week.ww

Forty-two systemically healthy patients with gingivitis (aged between 18–30) were included in the study. After baseline clinical measurements, all patients received initial professional prophylaxis (scaling – rubber-cup prophylaxis) and root planning as needed (Fig 1).

**Fig 2:**
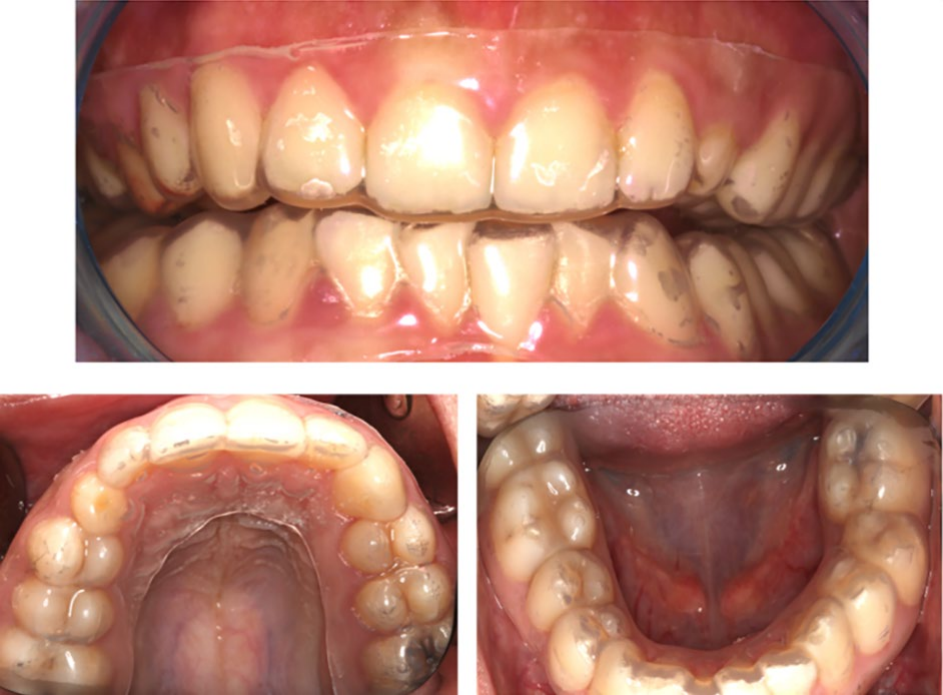
The test group (Zn group).

The patients were then randomised into the test group (Zn group) (n = 21) or control group (placebo stents without Zn) (n = 21) (Fig 2). The randomisation process was performed according to a computer-generated randomisation list. The allocation of patients to the groups was carried out by an independent researcher who was not involved in the clinical procedures or outcome assessments.

Stent fabrication steps:

Alginate impressions were taken from all patients, both maxillary and mandibular teeth.
The plaster models were poured. A vacuum thermoforming equipment (Biostar®️ V MachineType05, power: 230 V, 50/60 Hz500 W; working pressure: 0.5–6.0 bar) was used to prepare the customised stents.
A zinc-containing thermoplastic plate (ZinkH, tpe-based disc with 1–5% zinc additive, 2 mm thickness, Elemental, Belgium) for the test group and a blank plate (Erkoflex 125, ethylvinyacetate, 2 mm thickness, Erkodent, Australia) for the control group were placed on the models, which were stabilised on the machine’s platform. The zinc content used in the stents is as specified in the material data sheet provided by the manufacturer. Instead of a fixed concentration, it has been stated that the zinc content may vary between 1% and 5%. This range reflects the nominal variation in the composition of the material. Zinc is homogeneously distributed within the stent during the production process.
The materials were heated and formed on the models using standard operating pressure. After cooling the formed material for 2 min under pressure, the stents were removed from the machine and the borders of the stent covering the palatal, occlusal and buccal surfaces of the teeth were shaped to cover the mucogingival line.

Patients received oral hygiene instructions, including brushing and flossing. No rinses were used. They were also instructed on how to wear and remove the stents and not to chew while wearing them. They were asked to wear the stents for 4 weeks, for at least 12 h a day.

### Outcome Measures

One blinded, trained and calibrated examiner performed all clinical measurements using a periodontal probe (UNC-15 periodontal probe, Hu-Friedy, Chicago, ILHu-Friedy®) at baseline, 2nd, 4th, and 8th weeks.

#### GI

The primary outcome measure of the study was GI. The GI was measured at six sites per tooth, according to Loe & Silness. The mean GI was calculated by dividing the sum of all scores by the total number of surfaces examined.

0 – Normal gingiva;
1 – Mild inflammation with slight colour change, mild alteration of the gingival surface structure and no BOP;
2 – Moderate inflammation with oedema, redness, swelling and BOP;
3 – Severe inflammation with marked oedema and redness, ulceration and tendency to bleed spontaneously.

#### PI

The PI was measured after discolouration with Mira-2-ton disclosing solution at the vestibular surfaces of the teeth according to the Turesky modification of the Quingley & Hein index. The mean PI was obtained by dividing the sum of all plaque scores by the total number of scored surfaces examined.

0 – No plaque;
1 – Separate spots of plaque at the cervical margin of the tooth;
2 – A thin, continuous band of plaque at the cervical margin;
3 – A band of plaque wider than 1 mm but covering less than one-third of the tooth;
4 – Plaque covering more than one-third and less than two-thirds of the crown;
5 – Plaque covering more than two-thirds of the crown.

#### BOP

The BOP was measured by using a periodontal probe at six sites per tooth and was recorded as (+) or (–).

PPD was measured from the gingival margin to the base of the pocket. PPD, GR, CAL and measurements were performed at the midbuccal aspect of the teeth, by a manual probe and were rounded up to the nearest millimetre. The patient and the dentist taking clinical indices were blinded to the study groups.

### Statistical Analysis

Variables were summarised as mean ± standard deviation and median (minimum-maximum), whereas categorical variables were represented as numbers and percentages. The normality of the distribution was established using the Shapiro–Wilk test. To compare continuous variables between two groups, the Mann–Whitney U test was utilised. Because the data were not normally distributed, the F1-LD-F1 design was used for longitudinal data analysis. The F1-LD-F1 model produces an ANOVA-style statistic for group, time, and group-time interactions. Since the data in the study did not show a normal distribution, the F1-LD-F1 statistical method, which is used for the non-parametric analysis of repeated measures data, was preferred. All statistical analyses were conducted using IBM SPSS 20 (Armonk, NY, IBM) and RStudio (RStudio Team, 2020). All tests were deemed statistically significant at the 0.05 level.

## RESULTS

**Fig 3:**
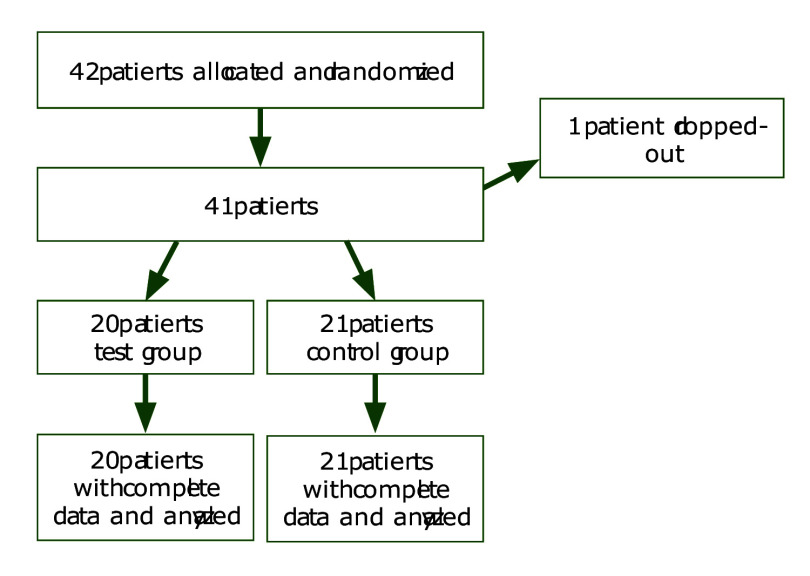
A flow diagram showing the enrollment and allocation of subjects involved in the study.

The flow chart of the study is shown in Figure 3. A total of 42 patients (Zn group: aged between 18 and 30, 11 female and 10 male) (control group: aged between 18 and 31, 10 female, 11 male) were treated. One patient in the study group did not attend the follow-up visits and was therefore excluded from the study. Finally, 41 patients completed the study. No side effects or complications related to the wearing, removal, movement, or fracture of the stents were reported. Patients were encouraged at each visit to adhere to the stent-wearing protocol. None of the participants reported discomfort or difficulty wearing the stents for 12 h per day. While we acknowledge that such extended daily use may not be feasible for all patient populations, our pilot data suggest that, in carefully selected and motivated individuals, wearing the stent for 12 h per day for 4 weeks was both feasible and well-tolerated.

The results of the primary outcome measure, GI is shown in Table 1. In the intragroup analysis, no statistically significant differences were observed at baseline; however, a statistically significant improvement was noted in both groups from baseline to the 8th week. The intergroup analysis showed that although no statistically significant difference was detected between the groups at baseline, GI scores were consistently and statistically significantly lower in the Zn group compared to the control group at all time points (P <0.001).

**Table 1:** The results of the primary outcome measure, GI. GI: gingival index, PI: plaque index, BOP: bleeding on probing, PPD: pocket depth, GR: gingival recession, AL: attachment level, SD: standard deviation (P <0.05).φ: Mann–Whitney U test, γ: Friedman test within groups between times.

	Time	Control (mean ± SD, median [min–max])	Treatment (mean ± SD, median [min–max])	P value
**GI**	T0	2.01 ± 0.67, 2(1–3)	1.99 ± 0.67, 2(1–3)	0.289
	T1	1.79 ± 0.68, 2(1–3)	1.64 ± 0.59, 2(0–3)	**<0.001**
	T2	1.78 ± 0.67, 2(1–3)	1.62 ± 0.6, 2(0–3)	**<0.001**
	T3	1.77 ± 0.67, 2(1–3)	1.61 ± 0.61, 2(0–3)	**<0.001**
	pγ	**<0.001**	**<0.001**	–
**PI**	T0	3.38 ± 0.94, 3(1–5)	3.4 ± 1.02, 3(1–5)	0.143
	T1	2.39 ± 0.84, 2(1–5)	2.45 ± 0.98, 2(0–5)	0.183
	T2	2.36 ± 0.84, 2(1–5)	2.41 ± 0.94, 2(0–5)	0.203
	T3	2.33 ± 0.84, 2(1–5)	2.36 ± 0.89, 2(0–5)	0.230
	pγ	**<0.001**	**<0.001**	–
**BOP**	T0	0.68 ± 0.47, 1(0–2)	0.7 ± 0.46, 1(0–1)	0.197
	T1	0.6 ± 0.49, 1(0–2)	0.59 ± 0.49, 1(0–1)	0.499
	T2	0.59 ± 0.49, 1(0–2)	0.58 ± 0.49, 1(0–1)	0.177
	T3	0.59 ± 0.49, 1(0–2)	0.58 ± 0.49, 1(0–1)	0.277
	pγ	**<0.001**	**<0.001**	–
**PPD**	T0	2.48 ± 0.99, 2(1–5)	2.46 ± 0.77, 2(1–5)	0.133
	T1	2.40 ± 0.94, 2(1–5)	2.37 ± 0.72, 2(1–4)	0.108
	T2	2.38 ± 0.93, 2(1–5)	2.37 ± 0.72, 2(1–4)	0.26
	T3	2.37 ± 0.92, 2(1–5)	2.37 ± 0.72, 2(1–4)	0.29
	pγ	**<0.001**	**<0.001**	–
**GR**	T0	0.03 ± 0.19, 0(0–2)	0.03 ± 0.18, 0(0–2)	0.229
	T1	0.03 ± 0.19, 0(0–2)	0.03 ± 0.19, 0(0–2)	0.258
	T2	0.03 ± 0.19, 0(0–2)	0.03 ± 0.19, 0(0–2)	0.258
	T3	0.03 ± 0.19, 0(0–2)	0.03 ± 0.19, 0(0–2)	0.258
	pγ	**0.308**	**>0.999**	–
**CAL**	T0	2.5 ± 0.99, 2(1–6)	2.48 ± 0.78, 3(1–5)	0.102
	T1	2.43 ± 0.94, 2(1–6)	2.4 ± 0.73, 2(1–5)	0.094
	T2	2.41 ± 0.93, 2(1–6)	2.4 ± 0.73, 2(1–5)	0.23
	T3	2.4 ± 0.92, 2(1–6)	2.4 ± 0.73, 2(1–5)	0.28
	pγ	**<0.001**	**<0.001**	–


PI and BOP scores are presented in groups, with PI and BOP scores at the 8th week showing a statistically significant reduction compared to baseline. However, intergroup comparisons revealed no statistically significant differences in PI and BOP scores at any time point.

At the baseline (T0), PPD and CAL scores were similar in both the control and Zn group (P >0.05). A statistically significant decrease was observed in both groups over time (P <0.001). However, no significant differences were found between the groups (P >0.05)

**Fig 4:**
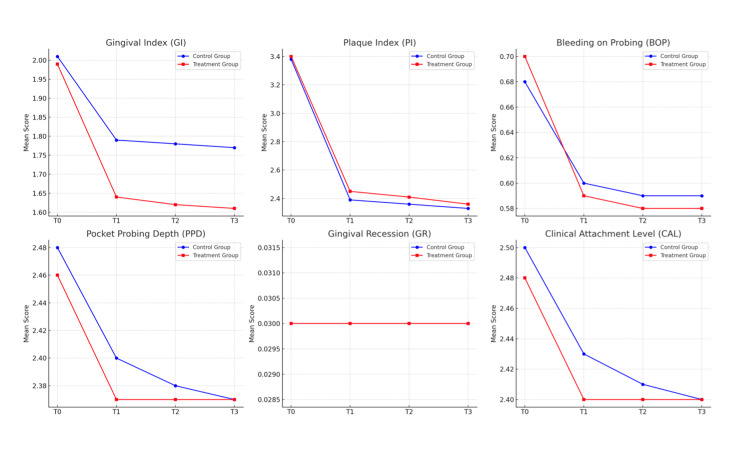
Evaluation and comparison of clinical outcomes between test and control groups over time.

There was no statistically significant change in gingival recession over time in both the control and Zn group (Fig 4).

## DISCUSSION

The main objective of this randomised controlled clinical trial study was to evaluate the short-term effects of Zn-containing stents as an adjunct to mechanical dental biofilm control on changes in gingival or bleeding indices. The results show that statistically significant improvement was observed in both groups in GI over time, and statistically significantly better GI scores were obtained in the group using Zn-containing stents compared to the control group, suggesting that Zn-containing stents may be a suitable adjuvant for preventing recurrence of gingivitis.

Therapeutic success or failure is influenced not only by the antibacterial effect of the medicine but also by the carrier system and the route of drug delivery.^15^ Antimicrobial agents can be effective both locally and systemically. Compared to systemic therapy, local delivery systems can administer medications continuously at higher concentrations and directly to the target region.^8^ Recent years have seen a surge in biopolymer-based wound dressing development for local applications.^35^ Zinc can be added to biopolymers due to its anti-inflammatory, antioxidant and anti-microbial characteristics.

Zinc is essential for preserving periodontal health because of its effects on oral soft tissues, both locally and immunologically. The clinical use of zinc is useful in treating various oral conditions, including periodontal inflammation, gingivitis, halitosis, and zinc deficiency, which has been linked to poor gingival and oral health. In the current study, PI scores decreased in both the study and control groups, possibly due to participants being instructed to brush their teeth twice daily for the whole study following the initial visit. Despite similar reductions in PI index scores, the additional usage of the Zn-stent led to a significantly higher reduction in mean GI scores over the 2-month follow-up period compared to the control group. GI scores are directly representative of gingival health and inflammation.^7,20^ This finding may be explained by the anti-inflammatory and wide-spectrum antibacterial effect of zinc in support of the literature.^5^ A significant improvement in GI, which directly reflects the primary pathological features of gingivitis, may be considered a meaningful indicator of clinical efficacy.

In the present study, the GI showed a statistically significant improvement in the group using zinc-containing stents, while no statistically significant intergroup difference was observed in BOP. Although both GI and BOP are widely used indicators of gingival inflammation, they reflect distinct aspects of the inflammatory process. GI is a composite score that accounts for visible clinical signs such as redness, oedema, and changes in surface texture, as well as bleeding on gentle probing. In contrast, BOP is a binary measure focused solely on the presence or absence of bleeding, reflecting the vascular response of the gingival tissue. The observed improvement in GI, despite the lack of change in BOP, may indicate that the zinc-containing stent had a greater impact on surface-level inflammatory signs than on deeper vascular reactivity. Given that GI directly reflects the primary pathological features of gingivitis, a statistically significant improvement in this parameter alone may be considered a meaningful indicator of clinical efficacy in this context.

Saliva contains a lot of antioxidants.^9^ Endogenous and exogenous antioxidants stabilise oxidative stress resulting from pathogen-induced inflammation.^27^ There are numerous studies showing that antioxidants are beneficial in the treatment of gingivitis.^29^ Zinc helps in the stability of cell membranes and provides antioxidant activities. In 45 periodontitis patients, Moreno et al performed non-surgical periodontal treatment on a control group and non-surgical periodontal treatment plus 500 mg magnesium oxide and 50 mg zinc gluconate for oral supplementation for 30 days on a study group. They found that zinc gluconate and magnesium oxide can be used as an adjunct to non-surgical periodontal therapy to assist the remission of periodontal disease by controlling the reduction of oxidative indicators and increasing the activity of antioxidant enzymes.^1^

The present study was conducted on young and healthy individuals. However, a large portion of the population, namely elderly individuals, hospitalised patients, or those with physical or mental disabilities, were excluded from the study. In these populations, mechanical plaque control is often inadequate due to limited manual dexterity or cognitive impairments. Similar to the role of electric toothbrushes in enhancing plaque control in patients with poor oral hygiene practices,^34^ zinc-containing stents may support gingival health by providing antimicrobial and anti-inflammatory effects. Therefore, even in individuals who are unable to perform optimal mechanical plaque removal, these stents could contribute to improved periodontal outcomes. Further studies are needed to specifically investigate this hypothesis in these at-risk groups.

Patient compliance is a critical factor for treatment success, and the feasibility of long-term intraoral appliance use has been well demonstrated in dentistry. For instance, orthodontic aligner therapy requires patients to wear aligners for approximately 20 to 22 h per day to achieve effective tooth movement.24 Studies have shown that, when patients are adequately informed and motivated, adherence to such prolonged usage is generally high. In this context, the prescribed wearing duration in our study – 12 h per day – is relatively more flexible and likely to be more compatible with patients’ daily routines. Indeed, no complications or compliance-related issues were reported during the study period, supporting the feasibility of the proposed protocol.

The other limitations of the study are as follows. The follow-up period was limited to 8 weeks, preventing an assessment of the long-term efficacy of Zn-containing stents in preventing gingivitis recurrence. Additionally, the study was conducted on systemically healthy young adults; therefore, the findings may not be generalisable to older populations or individuals with systemic conditions affecting periodontal health. Although the sample size of 41 participants is consistent with similar randomised controlled trials on gingivitis, the limited cohort size restricts the generalisability of our findings. Furthermore, lifestyle parameters that may potentially affect gingival inflammation and immune response, such as diet (eg, antioxidant intake), stress levels, and sleep patterns, were not assessed or controlled for in this study. Moreover, it should be considered that both compliance with stent usage and the duration of use were based solely on patients’ self-reported statements, and the actual wearing time was not objectively measured. This represents a methodological limitation, as self-reported data may be subject to recall bias or overestimation. Future studies involving larger and more diverse populations, objective measurement of stent compliance, longer follow-up periods, and consideration of these lifestyle factors are necessary to confirm and extend these results.

Despite these limitations, the clinical relevance of this study lies in demonstrating that the adjunctive use of zinc-containing stents can statistically significantly improve gingival health in patients with plaque-induced gingivitis. The findings provide additional evidence supporting the development of site-specific and controlled-release drug delivery systems in periodontal therapy. Given the growing interest in personalised and minimally invasive dental treatments, zinc-containing stents may represent a promising adjunctive treatment option for improving clinical outcomes in individuals who are unable to achieve optimal mechanical plaque control.

## CONCLUSIONS

In conclusion, the findings of this study suggest that Zn-containing stents provide additional benefits in reducing gingival inflammation beyond mechanical plaque removal alone. The significant reduction in GI scores in the Zn-stent group highlights the potential of Zn-containing biomaterials as an adjunctive approach in gingivitis management. Further long-term studies are warranted to explore the broader clinical applications of Zn-containing stents in periodontal therapy. A comprehensive evaluation of the results indicates that Zn-containing stents may provide significant clinical benefits as a novel and potentially effective adjunctive approach in periodontal therapy.

### Acknowledgements

#### Ethics approval and consent to participate

The study, which was conducted following the ethical rules of the Declaration of Helsinki, was approved by the Ethics Committee of Çukurova University Faculty of Medicine (5 May 2023/133/55).
